# The Effect of Music Therapy and Aromatherapy with Chamomile-Lavender Essential Oil on the Anxiety of Clinical Nurses: A Randomized and Double-Blind Clinical Trial

**DOI:** 10.25122/jml-2019-0105

**Published:** 2020

**Authors:** Somayeh Zamanifar, Mohammad Iraj Bagheri-Saveh, Aram Nezakati, Rozhin Mohammadi, Jamal Seidi

**Affiliations:** 1. Student Research Committee, Kurdistan University of Medical Sciences, Sanandaj, Iran; 2. Clinical Care Research Center, Research Institute for Health Development, Kurdistan University of Medical Sciences, Sanandaj, Iran; 3. Department of Medical Surgical Nursing, Besat Hospital, Kurdistan University of Medical Sciences, Sanandaj, Iran; 4. Department of Midwifery, Besat Hospital, Kurdistan University of Medical Sciences, Sanandaj, Iran

**Keywords:** Music, Aromatherapy, Anxiety, Nurses

## Abstract

Nurses may be anxious due to critical and emergencies, and anxiety can affect their professional performance. Non-pharmacological interventions, as a safe method, can reduce anxiety.

This study aimed to determine the effect of music therapy and aromatherapy with chamomile - lavender essential oil on the anxiety of clinical nurses.

This was a randomized, double-blind clinical trial. One hundred twenty nurses from clinical wards of Besat Hospital in Sanandaj, Iran, were selected between 2018 and 2019 by purposeful sampling. The samples were randomly assigned to three groups with different interventions, namely music therapy, both aromatherapy with chamomile-lavender essential oil and music therapy, and aromatherapy with chamomile-lavender essential oil, along with a control group as well. The anxiety of nurses was measured based on the Beck Anxiety Inventory before the intervention and after three work shifts. The data were analyzed using the SPSS v.22 software. One-way ANOVA was used to test the hypotheses.

The findings showed that the mean scores of the anxiety of nurses after the intervention in the three groups namely the group for which music therapy was applied, the group for which aromatherapy with chamomile-lavender essential oil was used and the group for which both music therapy and aromatherapy with chamomile-lavender essential oil were applied, were (39.28), (37.82) and (40.03), respectively. Therefore, the obtained mean score of each group was significantly lower than that of the control group (56.08) (p < 0.0001).

The results showed that the interventions of music therapy and aromatherapy with chamomile-lavender essential oil could reduce the anxiety of nurses. Therefore, it is recommended to use music therapy and aromatherapy with chamomile-lavender essential oil to reduce the anxiety of nurses in the clinical setting.

## Introduction

Anxiety is one of the major challenges for nurses in the clinical care (1). Nurses experience Anxiety is one of the significant challenges for nurses in clinical care [1] and nurses experience different levels of anxiety during care for various reasons [2]. The level of anxiety in nurses’ working environments in the literature is different. 43.4% of Chinese nurses had anxiety [3]. The level of anxiety in younger nurses higher in India, and the prevalence of anxiety in Indian nurses was 40% [4]. Some factors, such as work shifts and the critical condition of patients, cause the anxiety of nurses [5]. Anxiety is defined as “an excessive and uncontrollable worry”, which often interferes with daily functioning, and affects the mental ability to make appropriate decisions [6]. The anxiety of nurses increases the probability of error at work [7]; therefore, the use of safe and non-pharmacological interventions to reduce the anxiety of clinical nurses is essential. Non-medicinal methods such as music therapy [8] and aromatherapy have been mentioned in the literature to control the anxiety of nurses [7].

Aromatherapy is the use of aromatic oils, which makes people relax and reduces anxiety by stimulating the olfactory system and releasing nerve mediators [9]. Lavender is one of the most popular aromatic oils in aromatherapy and has anxiolytic effects [10]. In a study conducted by Fernandez et al., the inhalation of the essential oil of lavender reduced anxiety in nursing students [9].

Another medicinal plant used in aromatherapy is chamomile [11]. Chamomile, whose mechanism involves increasing cortisol secretion, reduces anxiety [12]. Essential oils can be combined for therapeutic purposes and greater effectiveness [13]; for example, in a study, Hashemi and Faghih used a combination of rose and lavender essential oils to investigate the hemodynamic status of nursing students [14]. The chamomile-lavender essential oil is one of the essential oils used to reduce the anxiety of patients. In another study, aromatherapy with chamomile-lavender essential oil reduced the anxiety of patients with coronary disorders [15]. In addition to aromatherapy, another non-pharmacological way to control anxiety is music therapy [16]. Music therapy is an inexpensive and non-invasive treatment, which can be used in conjunction with other complementary therapies [17]. Music reduces the sensation of pain and anxiety by distracting and reducing the focus of people on anxious stimuli [16]. Gramaglia et al. found in a review study that in more than 40 studies, music therapy reduced the anxiety of involved patients [18].

In addition to emphasizing the literature on the reduction of anxiety with non-pharmacological interventions [19], according to the principal researcher's experience, nurses in the clinic were looking for appropriate strategies to reduce anxiety. On a case-by-case basis, some nurses used music on the phone and even used anxiolytic drugs in some cases, so the principal researcher decided to provide some practical scientific solutions. Relating nurses’ experiences of anxiety and affective Factors, Gardiner and Sheen found that graduate nurses need support in order to reduce their anxiety [19]. Music therapy and aromatherapy are convenient and cost-effective ways to control the anxiety of nurses, which do not require special equipment and are easily operational. One of the other necessities of this study was the lack of a study to evaluate the effect of music therapy combined with the inhalation of chamomile-lavender essential oil on the anxiety level of nurses. Therefore, this study was conducted to determine the effect of music therapy and aromatherapy with chamomile-lavender essential oil on the anxiety of nurses in Besat Hospital of Sanandaj, Iran.

## Material and Methods

### Aim

The purpose of this clinical trial was to compare the anxiety levels of nurses in four groups with different interventions, namely music therapy, aromatherapy with chamomile-lavender essential oil, the combination of music therapy and aromatherapy with chamomile-lavender essential oil, and a control group.

### Design

The study was a randomized and double-blind clinical trial conducted between 2018 and 2019. It continued in August, September and October. The study population consisted of 120 nurses working at Besat Hospital in Sanandaj, Iran. Inclusion criteria included people with a healthy sense of smell, absence of allergic diseases, respiratory diseases and asthma, no pregnancy and lactation, work shifts (morning, evening and night), no leave or sick leave during the study (overnight offs are not part of the leave), willing to volunteer for the study, lack of thyroid diseases or mental illness including anxiety, lack of anxiolytics and analgesics during the study, lack of accidents and life crises (traffic accidents, deaths of relatives, divorce) in the last six months, not using perfume and cologne during the study, high interest in listening to music and obtaining a minimum score of 8 for the Beck Anxiety Inventory, participation of each nurse in three consecutive days (morning, evening, and night shifts). Exclusion criteria included allergy to essential oils, unwillingness to cooperate in continuing music listening and aromatherapy, and critical clinical status.

### Setting

The study was conducted at Besat Hospital in Sanandaj, Iran. The research setting was represented by the wards of Besat Hospital in Sanandaj, Iran. The wards included Adult Emergency, Pediatric Emergency, Adult ICU, Pediatric Internal, Pediatric Infectious, Neonatal ward, Pediatric ICU, Oncology, Internal ward, and Infants ward.

### Sample

The samples were selected by a purposeful sampling based on the inclusion criteria. Then, the 120 samples were allocated into three intervention groups and one control group based on random stratified sampling. In this study, each ward of the mentioned hospital was considered as one stratum. The number of samples per strata was determined according to the size of the strata, considering the total size of the community and the size of each stratum. Given the 10 clinical wards and the sample size for each group of 30 persons, 12 persons of each stratum were randomly assigned to a group by random numbers from one to four (the number of groups) without substitution, in the form of 3 persons in each group. Therefore, for each section, as a single layer, cards were assigned to groups 1 for music intervention, 2 for aromatherapy intervention, 3 for aromatherapy and music combination intervention, and 4 for the control group. Based on randomly selected cards, sampling was continued until completing the sample size in each layer.

In this study, the principal researcher and nurses know the intervention, but the co-researcher (data collector) and statical advisor (analyzer) were not responsible for the interventions regarding the type of blind intervention. In this study, none of the samples were excluded until the end of the intervention, so the sample number did not fall.

### Intervention

The intervention in the aromatherapy group was the inhalation of the chamomile-lavender mixed aroma. 1.5% chamomile-lavender essential oil was prepared from Zardvand Pharmaceutical Company. Then, at Shahid Beheshti School of Pharmacy, after confirming the scientific name, distillation with water was applied for this essential oil and was then diluted in sesame oil by 5%. In the aromatherapy group, for each sample, three drops of chamomile-lavender essential oil were poured on a pre-prepared no absorbable pad and were placed 20 cm from the nose [14] for 20 minutes [20]. This intervention was performed during three consecutive shifts and during employees’ rest time. The intervention in the music therapy group included the selection of conventional and favorite music (traditional, pop, and classic) according to the interest of the samples, and listening to them using headphones. This intervention was performed for 20 minutes [14] per shift, during three consecutive shifts and at the employees’ rest time. In the third group, the inhalation of chamomile-lavender mixed aroma and music playing were performed simultaneously in three consecutive shifts. The control group did not receive any intervention.

### Data collection

The method of data collection was that the principal researcher obtained the demographic characteristics of the nurses before entering the study. If each participant (nurse) is eligible to participate in the study and written consent has been received from him/her, the second part, the pre-test anxiety questionnaires were given to the nurses by the first research assistant, and data were collected by the principal researcher. After the random assignment of the samples to the intervention and control groups and after the intervention (at the end of three consecutive shifts) in the intervention groups, also at the end of study in the control group (at the end of three consecutive shifts), the anxiety assessment questionnaires were given to the nurses by by the second researcher assistant again and were collected after completion. The data were then submitted to the principal researcher for analysis.

### Anxiety measuring tool

In this study, the Beck Anxiety Inventory (BAI) was used to measure the anxiety of nurses. BAI is one of the most commonly used anxiety inventories, which precisely measures the severity of clinical symptoms of anxiety in persons; this questionnaire was introduced in 1990 by Beck et al. [21]. Subsequent studies confirm the applicability and validity of BAI in different situations [22, 23]. Omidi Hosein Abadi et al. used this tool to assess the anxiety of nurses in their study, and a reliability coefficient of 0.92 was estimated using Cronbach's alpha test [5]. The questionnaire contains 21 questions; each question is scored in a four-part range from zero to three. Each of the questions of the questionnaire describes one of the common symptoms of anxiety (mental, physical, and phobic). The total score ranges from 0 to 63. A score of 0 to 7 means none or the least amount of anxiety, a score of 8 to 15 means mild anxiety, a score of 16 to 25 means moderate anxiety, while a score of 26 to 63 shows severe anxiety [22]. In this study, the average anxiety score of each person was measured, and the mean anxiety scores were then compared in the four groups.

### Data analysis

The Chi-square test was used to investigate the frequency distributions of sex, marital status, educational level, and the number of children in the intervention and control groups. One-way ANOVA was used to examine the mean age distribution and to compare the level of anxiety in the intervention and control groups before and after the intervention. The Bonferroni post-hoc test was also used to examine the significant differences between the control group and the other groups. Data were analyzed by SPSS version 22 software.

### Ethical considerations

In order to begin the research, the ethics code IR.MUK.REC.1397.102 was obtained from the Ethics Committee of Kurdistan University of Medical Sciences. Then, the study was registered with the code number of IRCTID: IRCT20180923041106N1 at the Iranian Registry of Clinical Trials. Written consents were obtained from the study samples for voluntary participation in the study. The nurses were assured that the information collected would be confidential. Data were analyzed and reported without any identification. The samples could be excluded at any time.

## Results

The results of this study showed that demographic characteristics of the nurses, including sex, marital status, the number of children, educational level, and age were not significantly different in the intervention and control groups. The frequency distributions of demographic characteristics including sex (p = 0.595), the number of children (0.582), marital status (p = 0.825), educational level (p = 0.410) and the mean age (p = 0.977) showed no significant differences in the intervention and control groups (Table 1). The results of the one-way ANOVA showed that there was no significant difference between the mean and standard deviation of the anxiety of nurses in the four groups before intervention (p* = 0.990). The mean and standard deviation of the anxiety score of nurses, before the intervention, in the groups of music therapy, aromatherapy with chamomile-lavender essential oil, the combination of music therapy and aromatherapy with chamomile-lavender essential oil and control were 58.77 ± 13.14, 58.20 ± 15.02, 58.47 ± 10.68 and 57.70 ± 11.09, respectively. According to the results of the one-way analysis of variance, it can be concluded that there were significant differences between the post-intervention anxiety scores in the different groups (P <0.001). The difference between the pre-intervention anxiety and post-intervention anxiety was also significant in the four groups (p <0.05) (Table 2). The mean scores of the anxiety of nurses after the intervention in the groups of music therapy, aromatherapy with chamomile-lavender essential oil, the combination of music therapy and aromatherapy with chamomile-lavender essential oil were 39.28 ± 8.45, 37.82 ± 8.79 and 39.97 ± 9.38, respectively and, compared to the control group with the mean score of 51.37 ± 9.58, there was a significant decrease (p = 0.0001) (Table 2).

**Table 1:** The frequency distributions of demographic characteristics in the intervention and control groups.

Group Variable		Music therapy N (%)	Aromatherapy N (%)	Music therapy & Aromatherapy N (%)	Control N (%)	p-value
Sex	Male	4(30.8)	4(30.8)	1(7.7)	4(30.8)	P*= 0.595
Female	26(24.3)	26(24.3)	29(27.1)	26(24.3)
Material status	Single	12(30.0)	9(22.5)	10(25)	9(22.5)	p*= 0.825
Married	18(22.5)	21(26.3)	20(25)	21(26.3)
Educational level	Bachelor	30(26.8)	27(24.1)	28(25)	27(24.1)	p*= 0.410
MSc	0(0)	3(42.9)	2(28.6)	3(42.9)
Number of Children	No children	14(53.8)	24(64.9)	13(43.3)	14(53.8)	p*= 0.582
	One Child	4(15.4)	8(21.6)	7(23.3)	5(20.8)	
	Two Children	7(25.9)	5(13.3)	9(30)	4(16.7)	
Three Children	1(3.7)	0(0)	1(3.3)	1(4.2)
Age	mean	32.33	32.27	32	32.60	p**= 0.977
SD	±4.59	±4.66	±5.53	±5.83

Note: P*= the Chi-Square Test / P**= One-Way ANOVA Test.

**Table 2:** The mean value of anxiety before and after the intervention in the intervention and control groups.

Group Variable	Music therapy		Aromatherapy		Music therapy & Aromatherapy		Control		p-value
mean	SD	mean	SD	mean	SD	mean	SD
Anxiety before the intervention	58.77	±13.14	58.20	±15.02	58.47	±10.68	57.70	±11.09	p*= 0.990
Anxiety after the intervention	39.74	±8.45	37.83	±8.79	39.97	±9.38	51.37	±9.58	p*= 0.0001
Anxiety difference before and after the intervention	19.03	±12.25	20.36	±16.24	18.50	±12.59	6.33	±14.84	p*= 0.0001

Note: P*= One-Way ANOVA Test

The results of Levene's test for the equality of variances showed that the error variances of the groups were equal, and there was no reason for the heterogeneity of the error variances (p> 0.05) (Table 3). The p-value of the F test (p = 0.0001) showed that there was a significant difference between the groups in the mean anxiety after the intervention (Table 4).

**Table 3:** Levene's Test of Equality of error variances. Dependent Variable: Anxiety (after).

F	df1	df2	Sig.
0.925	3	113	0.431

**Table 4:** Binary Comparison Results.

Anxiety (after) Variable	mean difference	p-value
Aroma-music	-1.832	P***=0.999
Aroma-combined	-2.099	P***=0.999
Aroma-control	-13.59	P***=0.0001
Music- combined	-0.267	P***=0.999
Music-control	-11.767	P***=0.0001
Combined-control	-11.500	P***=0.0001

Note: P***= Bonferroni post-hoc test.

The Eta-squared or observed power showed that the group changes had more effect on the post-intervention anxiety compared to pre-intervention anxiety. The value of the modified explanation coefficient in the above model was 0.266, thereby indicating that the pre-intervention group and anxiety accounted for approximately 27% of the post-intervention anxiety changes, and another variance was influenced by factors and variables that were not studied in this study (Table 5).

**Table 5:** Tests of Between-Subjects Effects. Dependent Variable: Anxiety (after)

Source	Type III Sum of Squares	df	Mean Square	F	Sig.	Partial Eta Squared	Noncent. Parameter	Observed Power^b^
Corrected Model	3709.984^a^	4	927.496	11.52	0.000	0.292	46.117	1.000
Intercept	6041.703	1	6041.703	75.10	0.000	0.401	75.101	1.000
Anxiety. Before	305.150	1	305.150	3.793	0.054	0.033	3.793	0.488
Group	3453.770	3	1151.257	14.31	0.000	0.277	42.932	1.000
Error	9010.135	112	80.448					
Total	221974.00	117						
Corrected Total	12720.120	116						

Note: a. R Squared = .292 (Adjusted R Squared = .266); b. Computed using alpha = .05

## Discussion

Based on the results of this study, it was found that non-pharmacological interventions in the intervention groups, including music therapy, chamomile-lavender aromatherapy, and chamomile-lavender aromatherapy and music therapy simultaneously, reduced the anxiety of nurses significantly compared to the control group. Davis et al. reported that aromatherapy massage with music significantly reduced anxiety levels. However, there was no significant difference in the occupational stress level at 12 weeks after the intervention [24]. The results of this intervention were in line with the current study. The results of a study by Pradopo et al. showed a significant decrease in dental anxiety of pediatric patients after the music therapy and aromatherapy intervention (p <0.05) [25]. The results of this study confirm the results of the present study. The findings of the study conducted by Lee et al. in 2017, showed that music therapy and aromatherapy were effective in reducing the anxiety of patients undergoing mechanical ventilation [26]. The results of this study were similar to those of the present study. Xiao et al. also reported that music therapy and aromatherapy reduced the anxiety of breast cancer patients [27], which was consistent with the results of the present study. Onishi et al. found that complementary treatments, including relaxing music, aroma foot bathing, and resting on the bed, were effective in decreasing the stress of nurses in hospitals [28].

According to the results of a recent study, music therapy was effective in reducing the anxiety of nurses. Heidari et al. reported in their study that music therapy reduced the anxiety of patients undergoing coronary artery bypass graft surgery [8]. The results of this study were similar to those of the current study, except that the samples were different. The results of a study by Le Danseur et al. showed that music therapy had an impact on reducing the anxiety of stroke patients during rehabilitation [29]. In another study, Toker et al. concluded that music therapy had no effect on the anxiety of pregnant women with pre-eclampsia [30]. The difference between this study and the current study was in the characteristics of the samples, thereby indicating that the effect of music therapy on patients and nurse anxiety may vary since music therapy reduced the anxiety of nurses in the present study.

As the results showed, the anxiety of nurses decreased significantly after the inhalation of lavender-chamomile essential oil. In this regard, Cho and et al. found that aromatherapy with chamomile and lavender essential oils improved the anxiety of ICU patients [15]. In a systematic review, Li et al. found that aromatherapy and massage had a positive effect on reducing the stress of nurses [7]. The results of a study by Tabaraei et al. indicated the effect of the inhalation of chamomile essential oil in reducing the anxiety of candidate patients for endoscopic procedures [31]. These studies confirm the results of the present study, except that the characteristics of the samples were different. In their study, Heidari-Fard et al. reported that aromatherapy with chamomile essential oil reduced the anxiety of women during childbirth [32]. Donaldson et al., in their study results, reported that aromatherapy had no effect on the anxiety of nurses [2], which is inconsistent with the present study. This contrast may be due to the length of the study and the type of essential oil used. In the present study, a combination of music therapy and aromatherapy with chamomile-lavender essential oil was used to reduce the anxiety of nurses, but in their study, only aromatherapy was used for the intervention.

The results of other studies showed that music and aromatherapy were used in different combinations for different samples, whereas in the present study, inhalation of lavender chamomile and music therapy for nurses is a novelty. One of the limitations of this study was the occurrence of situational problems and mental issues for nurses during the research, which was left out by the researchers. Also, due to the rotation of the work shifts of nurses, sampling in three consecutive shifts was a problem in the study. The findings of this study indicated the effect of music therapy and aromatherapy with lavender-chamomile essential oil in reducing the anxiety of nurses. Therefore, this method is recommended as a safe method in work environments for nurses.

## Conclusion

The results of this study showed that music therapy and aromatherapy with chamomile-lavender essential oil had an effect on reducing the anxiety of nurses, which was significant compared to the control group. On the other hand, music therapy and aromatherapy had a similar effect on reducing the anxiety of nurses since anxiety is one of the nursing outcomes, and reducing the stress and anxiety can increase the level of accuracy and attention and improve the quality of nurses’ work. Then, music therapy and aromatherapy as complementary therapies can be used as an independent nursing intervention to improve nurses’ performance.

## Acknowledgments

This article is the result of a Master's thesis in Internal Surgery Nursing, entitled “Investigating the effect of music therapy and aromatherapy with chamomile-lavender essential oil on the severity of fatigue and the anxiety of nurses working at Besat Hospital in Sanandaj”, which was approved by the Ethics Committee of Kurdistan University of Medical Sciences, Sanandaj, Iran with the code IR.MUK.REC.1397.102 on 05-09-2018. This article was carried out without any financial support from the University and the Honorable Deputy of Research and Technology of Kurdistan University of Medical Sciences, Sanandaj, Iran. We also appreciate the Student Research Committee, Honored Personnel of Besat Hospital, Research Associates, Honorable Professors and all members of the Faculty of Nursing and Midwifery of Kurdistan University of Medical Sciences for assisting us in this research.

## Conflict of Interest

The authors confirm that there are no conflicts of interest.
